# Evaluation of Ahmed glaucoma valve implantation through a needle-generated scleral tunnel in Mexican children with glaucoma

**DOI:** 10.4103/0301-4738.67039

**Published:** 2010

**Authors:** Oscar Albis-Donado, Félix Gil-Carrasco, Rafael Romero-Quijada, Ravi Thomas

**Affiliations:** Asociación Para Evitar la Ceguera en México, Departamento de Glaucoma, México DF; 1 Queensland Eye Institute and University of Queensland, Brisbane, Australia

**Keywords:** Ahmed glaucoma valve, children, glaucoma, glaucoma drainage implants

## Abstract

**Purpose:**

To evaluate the results and extrusion rates of the Ahmed glaucoma valve (AGV) implantation through a needle-generated scleral tunnel, without a tube-covering patch, in children.

**Materials and Methods:**

A retrospective review of the charts of 106 Mexican children implanted with 128 AGVs operated between 1994 and 2002, with the needle track technique, at our institution, with at least six months follow up was done. Main outcome measures were intraocular pressure (IOP) control, tube extrusions or exposure and other complications.

**Results:**

Kaplan-Meier analysis demonstrated a 96.9% survival rate at six months, 82.4% at one year, 78.7% at two years, 70% at three years and 41.6% at four years. Total success at the last follow-up (IOP between 6 and 21 mm Hg without medications) was achieved in 30 eyes (23.5%), 58 eyes (45.3%) had qualified success (only topical hypotensive drugs) and 40 eyes (31.3%) were failures. The mean pre- and post-operative IOP at the last follow up was 28.4 mmHg (SD 9.3) and 14.5 mmHg (SD 6.3), respectively. No tube extrusions or exposures were observed. Tube-related complications included five retractions, a lens touch and a transitory endothelial touch. The risk of failure increased if the eye had any complication or previous glaucoma surgeries.

**Conclusion:**

Medium-term IOP control in Mexican children with glaucoma can be achieved with AGV implantation using a needle-generated tunnel, without constructing a scleral flap or using a patch to cover the tube. There were no tube extrusions, nor any tube exposures with this technique.

Young patients present some of the most challenging glaucomas. They tend to rapidly repair surgical fistulas and the balance between intraocular pressure (IOP) control, wound modulation and complications is hard to achieve. Mitomycin C-enhanced trabeculectomies provide better IOP control, but are at risk of major complications like blebitis, endophthalmitis and bleb-leaks.[1–4] We abandoned mitomycin C use in this age group after we found a 10% endophthalmitis rate over five years in this age group (unpublished data).

Glaucoma implants in children were first reported by Molteno in 1973.[5] Reports with different types of implants in children show a relatively good control of IOP, but complications, such as hypotony, loss of light perception, choroidal detachment and phthisis are high, especially with non-valved implants.[6–10] The experience with the Ahmed glaucoma valve (AGV) in children also includes complications such as transient hypotony, rare persistent choroidal detachments and even loss of light perception, albeit unrelated to the implant *per se*.[11–13] Additional potential complications, especially in children with implants, are either tube exposure and / or extrusion.[12,14,15]

We have reported a scleral graft-free tube insertion technique using a long scleral tunnel created with a needle.[16,17] Similar techniques without the need for covering the tube with a patch have reported excellent safety as well as faster operating time.[18,19] Avoiding a scleral graft also maintains a normal angle between the conjunctiva and the cornea, reducing the risk of dellen, pain, foreign body sensation and bleb leaks and also allays concerns about prion transmission.[20] We present the results of our graft-free technique in Mexican children with refractory glaucomas operated on or before the age of 16 years.

## Materials and Methods

We performed a retrospective chart review of patients operated at our institution for refractory glaucomas before their sixteenth birthday using AGV models S2 or S3. The operations were performed between 1994 and 2002, using the graft-free technique developed by one of the authors.[16,17] Indications for surgery included previous failed procedures or associated eye conditions that increased the risk of failure of filtering or angle surgeries.

The reproducible surgical technique without the use of a scleral graft patch routinely used for the past 15 years has been described elsewhere.[16,17] In brief, a fornix-based conjunctival flap was made in the designated quadrant, and then the valve was primed with balanced salt solution (BSS) and fixated 8 to 10 mm behind the limbus with 7-0 silk. A scleral tunnel initiated 3-4 mm from the limbus was constructed using a 22 or 23 G needle, bent as a ‘Z’ to avoid obstruction from the eyelids, brow or lid speculum. Viscoelastic injection prior to or after tube insertion was not routine in those days, although we now commonly mount the needle with viscoelastic, to fill the anterior chamber, before inserting the tube.

The needle was passed bevel-up under the episclera, in a tangential direction. At the limbus the direction was abruptly changed to make the tunnel parallel to the iris, attempting to enter through the trabecular meshwork. The tube was then trimmed to create a 30-45° bevel and inserted through the tunnel into the anterior chamber. The conjunctiva was closed using a 7-0 absorbable suture for small children or the same 7-0 silk for older children. The post-operative regimen included steroid drops in a reducing dose for three months, antibiotic drops for two weeks and a cycloplegic for the first month.

For our study, the primary outcome measure was IOP control. Measurements under anesthesia were taken soon after induction using applanation (wherever possible) or Schiotz, by consultants or trained glaucoma fellows, until the child could cooperate for Perkins or Goldmann applanation tonometry without sedation.

Complete or total success was defined as a post-operative IOP between 6 and 21 mmHg, without additional topical or systemic medications. If such IOP control required the use of medications, it was defined as a qualified success. Failure was defined as the inability to achieve complete or qualified success as well as persistent hypotony (IOP under 6 mmHg), further glaucoma surgery including removal of fibrous tissue around the valve, removal of the tube and loss of light perception. One elevated IOP was enough to indicate failure if additional surgeries were undertaken, but not if medical treatment was tried first. The need for tube repositioning was not considered a failure if the other criteria for success were met.

The secondary outcome measure was the tube extrusion / exposure rate. We also reported complications, their impact on IOP control and tube extrusions / exposures, plus the impact of the procedure and the disease on visual acuity. The variables studied included age at the time of surgery, sex, diagnosis, previous procedures, medications, visual acuity, IOP before and at different time-points after surgery, IOP at last follow-up, AGV location, time to failure, training level of the surgeon, follow-up time and complications.

Data were collected and analysed using SPSS version 15. Means for continuous variables were compared among the different groups using ANOVA. Associations between categorical variables were studied with the Chi-square test; Fisher’s exact test was used when fewer than five cases were present at a given category, and we additionally computed the relative risk and confidence intervals for these data. Multivariate models were constructed to determine the effect on the final IOP and number of medications at the last follow-up. As both eyes of 20 patients were included in the analysis, generalised estimating equations (GEE) were used to adjust for dependencies. Forward stepwise selection of covariates and factors, significant in the simple analysis, was used for the final multivariate model. Survival analysis was performed using the Kaplan-Meier life-Table method, using the log-rank test for comparing survival times between groups.

## Results

During the study period 204 eyes of 167 patients were operated using this technique. Seventy-one eyes of 56 patients had less than six months follow-up and were excluded. At the last examination of these 71, 26 (36.6%) had failed (21 loss of IOP control, three lost light perception and two had been eviscerated: one for persistent pain and the other for persistent choroidals), three (4.2%) had qualified success and 42 (59.2%) had total success before being lost to follow-up. Four patients (four eyes) with incomplete charts and one patient (one eye) whose eye was enucleated due to retinoblastoma were also excluded.

The remaining 128 eyes of 106 patients were analysed. There were 62 right eyes (48.4%) of 106 patients; 65 (61.3%) were male and the age ranged from three months to 15 years (mean 7.58 years, SD 4.66 years). The follow-up ranged from six to 96 months (eight years), with a mean of 25.73 months (SD 20.47).

AGV implant surgeries were performed by the senior author (FGC) in 57 eyes (44.5%), by associate professors in 50 eyes (39.1%) and by glaucoma fellows in the remaining 21 eyes (16.4%). The AGV was placed in the superotemporal (ST) quadrant in 119 eyes (93%) and in the inferotemporal (IT) quadrant in the remaining nine (7%).

The most frequent primary diagnosis was congenital glaucoma (43 eyes; 33.6%) followed by developmental glaucoma, (32 eyes; 25%). Secondary glaucomas comprised the largest group of 53 eyes (41.4%), 16 eyes associated with trauma (12.5%) and 17 with aphakia (13.3%). The list of all the diagnoses and their success rates are detailed in Table 1. Multivariable analysis did not reveal any association between the type of glaucoma and final IOP or number of medications. There was a significant difference in the mean and median survival times, but the follow-up period in the developmental glaucomas was shorter; they began receiving AGVs at a later time at our service.

**Table 1:** Types of glaucoma and their success rate

Diagnosis	Eyes (n)	%	Successful n (%)	Median Survival
Congenital Glaucoma	43	33.59	27 (62.8)	60 months*
Developmental Glaucomas	32	25	20 (62.5)	25 months
Axenfeld-Rieger	12	9.38	7 (58,3)	
Other AS dysgenesis	12	9.38	8 (66.7)	
Peter’s Anomaly	2	1.56	0	
Sturge–Weber	2	1.56	2 (100)	
Microspherophakia	1	1.56	1 (100)	
Oculodentodigital syndrome	2	1.56	1 (50)	
Aniridia	1	0.78	1 (100)	
Secondary Glaucomas	53	41.4	41 (77.4)	60 months
Traumatic	16	12.5	12 (75)	
Aphakic	17	13.28	13 (76.5)	
Uveitic	8	6.25	4 (50)	
Pars Planitis	5	3.91	3 (60)	
Idiopathic	3	2.34	1 (33.3)	
Vogt-Koyanagi-Harada	0	0	0 (0)	
Post-Penetrating Keratoplasty	5	3.91	5 (100)	
Steroid related	4	3.13	4 (100)	
Retinal Surgery (1 with ROP)	3	2.34	3 (100)	
Pseudophakic	0	0	0	
Neurofibromatosis	0	0	0	
Total	128	100	88 (68.75)	54 months

AS: Anterior Segment, ROP: Retinopathy of Prematurity.

* Log-Rank test shows the difference in survival between the three main groups to be significant (*P* = 0.004)

**Figure 1:**
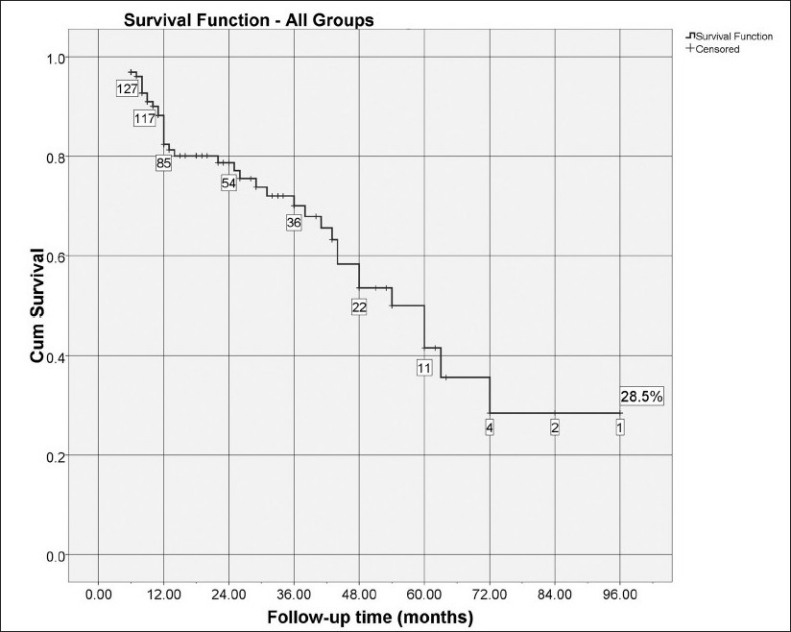
Cumulative probability of success through follow-up time (Kaplan-Meier survival curve). The number of cases remaining without failure events is shown in boxes

Kaplan-Meier analysis [Fig. 1] revealed a qualified and complete success rate of 96.9% at six months; 82.4% at one year, 78.7% at two years, 70% at three years and 41.6% at four years. From this point on less than 20 patients (15.75% of the original group) with more than four years of follow-up remained, and four more failures developed at 54, 60, 63 and 72 months of follow-up, making the cumulative survival drop to 28.5% at 96 months. The mean survival time for the whole group was 55.26 months (CI 46.6 – 63.9).

**Figure 2:**
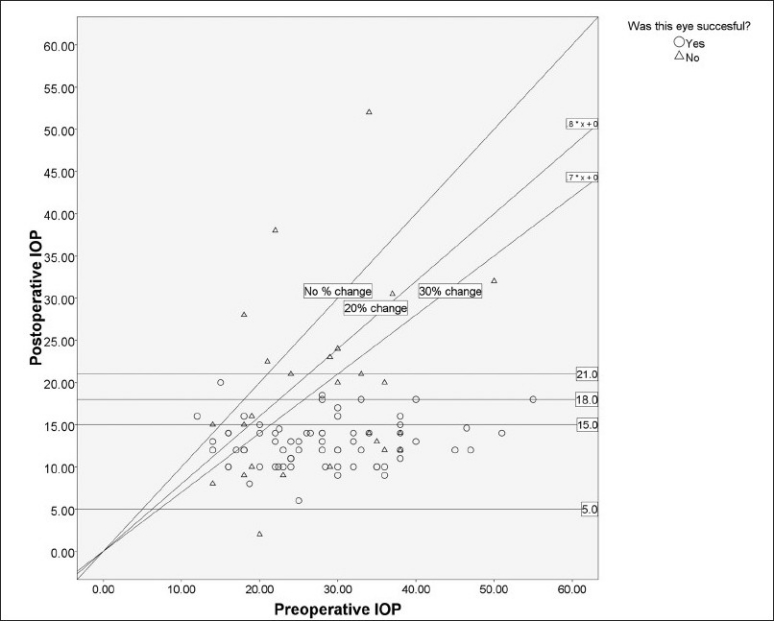
Scatterplot of preoperative intraocular pressure (IOP) versus postoperative final IOP. Each point represents one case. The oblique line represents the line of no change, and lines for IOP success criteria are also shown for reference. Additional lines for other success criteria are drawn for comparative purposes

The mean preoperative IOP was 28.46 mmHg (12-55, SD 9.36) on 1.03 medications (0-3, SD 0.955); oral acetazolamide was used for 18 patients (19 eyes). The IOP decreased to a mean of 14.5 mmHg (2-52, SD 6.34, n = 108) at the last follow-up on 1.03 medications (0-3, SD .955; only topical medications were required). The difference between preoperative IOP and final IOP was significant (*P* < 0.001, paired samples t test), but the difference in the number of medications required was not (*P* = 0.55). Fig. 2 shows a scatterplot of preoperative IOP versus final IOP. Table 2 shows the mean parameters according to the outcome.

**Table 2:** Demographics, medications, and intraocular pressure

	Success			Failure mean (SD)	Total mean (SD)	n
	Complete Success mean (SD)	Qualified Success mean (SD)	Global Success mean (SD)			
Age (years)	5.98 (4.79)	8.74 (4.38)*	7.78 (4.68)	7.1 (4.65)	7.58 (4.66)**	106
Pre-Operative Medications	0.93(0.78)	0.98 (0.93)	0.97 (0.88)	1.15 (1.07)	1.02 (0.94)	128
Pre-Operative IOP	27.9 (9.18)	28.09 (9.55)	28.02 (9.38)	29.5 (9.37)	28.46 (9.36)	113
IOP last control	12.05 (2.18)	13.15 (2.9)	12.86 (2.76)	18.58 (10.01)†	14.5 (6.34))‡	108
Last control Meds	0	1.55(0.68)*	1.02 (0.92)	1.05 (1.04)	1.03 (0.96)‡	128
Follow-up (months)	24.8 (18.51)	26.79 (22.23)	26.11 (20.95)	24.9 (19.6)	24.9 (19.6)	128

* Difference between complete success and qualified success significant at least at the 0.025 level or better.

** Difference between the complete, qualified, and failures significant at the 0.04 level.

† Difference between global success and failures significant at the *P* < 0.001 level.

‡ Difference between complete success, qualified success, and failures significant at the *P* < 0.001 level or better

At the last follow-up, success was achieved in 88 eyes (68.8%); complete success in 30 (23.40%) and qualified success in 58 (45.3%). The remaining 40 eyes (31.3%) comprised failures: loss of IOP control (23 eyes, 57.5% of failures) and loss of light perception (8 eyes, 20% of failures) were the most common causes. The details are shown in Table 3.

**Table 3:** Causes of failure and causes of NLP in Mexican children

	N	%	Notes
Causes of Failure			
Loss of IOP control	24	60	Bleb fibrosis removed in 20 eyes, four received an additional AGV after second fibrosis removal failure
NLP	15	37.5	See below
AGV removal (all were implanted with a new inferotemporal AGV)	4	10	Two corneal decompensations, one valve exposed after two retinal surgeries, one tube retraction
Eviscerated	0	0	Uveitic case
Valve extrusion	1	2.5	Post-traumatic case
Retinal detachment	1	2.5	
Total	40	100	
Causes of NLP			
Loss of IOP control	6	15	Glaucoma progression
Uncertain LP before surgery	3	7.5	Out of 11 eyes with uncertain LP before surgery (27.3%)
Retinal detachment	2	5	One post-traumatic, one Peter’s anomaly complicated 14 months after AGV during penetrating keratoplasty
Uveitis	1	2.5	Pars planitis
Retinal detachment and uveitis	1	2.5	Toxocariasis
Endophthalmitis	1	2.5	Developed at the site of the previous trabeculectomy with mitomycin C
Phthisis bulbi	1	2.5	Post-traumatic case, developed after retinal detachment surgery
Total NLP	15	100	

None of the patients in this series had tube extrusions. This was true even if the eyes with a follow-up of less than six months (total 204) were considered. The upper end of the confidence interval for this rate of zero extrusions in 204 eyes was 1.5%.

Forty-two eyes (32.8%) had undergone at least one surgery prior to their first consultation, of which 40 (41.2%) were glaucoma surgeries [Table 4]. The mean final IOP and mean number of topical medications to reach that IOP were not affected by this risk factor. Survival times and success rates were significantly affected by previous glaucoma surgeries, but not by other types of surgeries [Table 4]. Congenital glaucomas tended to have had more previous glaucoma surgeries and secondary glaucomas more non-glaucoma surgeries, but the type of glaucoma or its interaction with previous surgeries did not affect the outcome significantly.

**Table 4:** Types of surgeries done prior to AGV implantation and impact on Kaplan-Meier survival times (Mantel-Cox Log-rank test)

	Total		Mean survival (months)
	n	%	
Non-Glaucoma Surgeries			
Cataract	25	43.9	
Retina / vitreous	3	5.3	
Vitrectomy	7	12.3	
PK	4	7.0	
Other Surgeries	14	24.6	
Unknown*	4	7.0	
Total Non-Glaucoma	57	100.0	45.46**
Glaucoma Surgeries				
Trabeculectomy w/ mitomycin C	8	20	
Goniotomy	3	7.5	
Trabeculectomy	11	27.5	
Ciclodestructive	1	2.5	
Previous AGV	3	7.5	
Sclerostomy	1	2.5	
AGV removal	2	5.0	
Unknown*	11	27.5	
Total Glaucoma Surgeries	40	100.0	32.66†
Any previous surgery	49		38.88
No previous surgery	79		61.06‡

* Done at other institutions. AGV: Ahmed Glaucoma Valve, PK: Penetrating Keratoplasty.

** *P* = 0.878 compared to eyes not having previous non-glaucoma surgeries.

† *P* = 0.003 compared to eyes without previous glaucoma surgeries.

‡ *P* = 0.049 compared to eyes with any previous surgery

**Figure 3:**
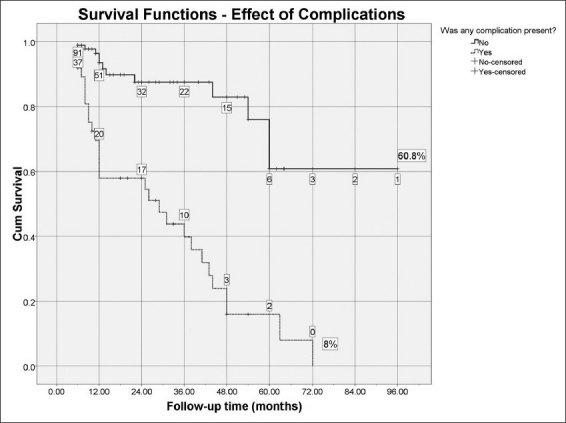
Cumulative probability of success through follow-up time comparing eyes without complications versus eyes with any complication (*P* < 0.001, Kaplan-Meier survival curve). The number of cases remaining without failure events is shown in boxes

Complications did, in fact, affect the outcome greatly. Any kind of complication at any time after surgery was present in 37 eyes (28.9%). Compared to 79 successful eyes (89.8%) of the 91 without complications, only nine (10.2%) eyes with complications were considered to be successful, (relative risk 6.84, 95% CI 3.56 – 13.13, *P* < 0.001). Kaplan-Meier analysis revealed a mean survival of 74.9 months in non-complicated eyes versus 30.4 months for eyes with any complication [*P* < 0.001, Fig. 3].

Bleb fibrosis leads to elevated IOP and failure by definition. Excluding eyes with such bleb fibrosis, 23 eyes (18%) had other types of complications and only eight (34.8% of 23) were considered successful, compared to 80 eyes (76.2% of the remaining 105) without complications (relative risk 4.12, 95% CI 1.0 – 8.9, *P* < 0.001). Kaplan-Meier analysis revealed a mean survival of 36 months for eyes with complications excluding bleb fibrosis versus 63.6 months for those without (*P* = 0.002). Multivariable analysis revealed a net effect of 1.47 mmHg greater than the final IOP on eyes with any kind of complications (*P* = 0.31). Complications and their effect on the outcome are listed in Table 5.

**Table 5:** Complications and their effect on the outcome

Complications	Failure n (% within complication)	Total n (% of 186)
Bleb fibrosis	13 (92.9)	14 (10.9)
Corneal decompensation	4 (57.1)	7 (5.5)
Flat anterior chamber	0 (0)	3 (2.3)
Retracted tube	3 (75)	4 (3.1)
Retinal detachment	2 (100.0)	2 (1.6)
Removal	0	0
Valve extrusion	2 (100.0)	2 (1.6)
Choroidal detachment	0	0
Pain	1 (100.0)	1 (0.8)
Endophthalmitis	1 (100.0)	1 (0.8)
Lens-tube touch	1 (100.0)	1 (0.8)
Transitory endothelial touch	0 (0.0)	1 (0.8)
Displaced valve	0	0
Total complications	27 (75)	36 (29.9)

Success rates for superotemporal (83 of 119, 69.7%) and inferiorly placed AGVs (five of 9, 55.6%) tend to favor the former site. Survival analysis showed a mean survival of 54.9 months (median 54 months) for superotemporal versus 49.56 months (median 60 months) for inferotemporal, but the difference was not significant (*P* = 0.163, Breslow). Despite this, complications were more frequent in the inferior location (six of 9, 66.7%) versus the superior location (31 of 119, 26.1%) and this difference was significant (*P* = 0.017); the interaction of these two variables did not affect the final IOP on multivariate analysis.

It was possible to determine the visual acuity before and after surgery in 107 eyes (83.6%). The acuity remained stable in 26 eyes (20.3%), improved in 46 eyes (35.9%), and worsened in 35 eyes (32.7%). There was a tendency for stabilization or improvement of vision in congenital glaucoma cases (28 of 33, 84.8%) as compared to secondary glaucomas (29 of 47, 61.7%) or developmental glaucomas (15 of 27, 55.6%), and this difference was statistically significant (*P* = 0.031).

Worsening of visual acuity was associated with a greater risk of failure, but this was not surprising, as loss of light perception was one of the criteria used to define failure (*P* = 0.023). Other analyzed risk factors such as training level of the surgeon, valve model (only 4 S3s, all successful), valve location, age, need of repositioning the tube any time after surgery (n = 6), use of eye massage to maintain IOP control, uncertain light perception before surgery, initial IOP greater than 40, initial visual acuity worse than 20/200 or counting fingers (CF) at 3 m, and the use of oral acetazolamide before surgery (n = 19) did not affect the success rates, survival times or final IOP (using ANOVA, Kaplan-Meier, and multivariate analysis, respectively).

The final multivariate model, using generalized estimating equations and forward stepwise selection of factors showed that any complication increased the likelihood ratio of failure to 1.78 (CI 0.73 – 2.84, *P* = 0.001) and previous glaucoma surgeries increased it to 1.2 (CI 0.24-2.18, *P* =0.016).

## Discussion

The present study shows that medium-term IOP control in Mexican children with glaucoma is feasible in many cases by the use of an AGV. Our results demonstrate that insertion of the tube using the needle-tunnel technique, without additional patching for the tube is safe and effective, both for IOP control and minimization of tube-related complications. It is noteworthy that none of the children, (even those excluded due to a shorter follow-up period) had tube extrusion or exposure, and none had persistent hypotony or flat anterior chamber as a cause of failure.[15]

Of course IOP control over several years means many different examiners, tonometers, and techniques, both with and without general anesthesia. This introduces an inherent bias, somewhat compensated by the fact that all children had the same chance for every technique and instrument, but not as good as if the study had been conducted prospectively.

A summary of the reported rates of tube extrusions with different types of patients and techniques is shown in Table 6. Even using the eyes excluded due to lower follow-up times, we found no tube extrusions in our series. The zero extrusions in 204 eyes were still compatible with a true rate of 1.5%, which was lower than the mean of most reports. We believe that the explanation for the lack of tube extrusions was related to a tighter fit between the tube and the tunnel, allowing for less tube movement and therefore less abrasion and micromovements, which could potentially lead to extrusion.[15,17] Another benefit of this technique was a flatter transition between the conjunctiva and the cornea, improving the lubrication of the limbus and avoiding complications such as dellen and painful blebs, which are more common with patch grafts.[21,22] No additional sutures were placed to fix the tube, as was recommended for the scleral flap technique, to avoid extrusions, as the needle tract provided enough immobility.[23,24]

**Table 6:** Extrusion rates from 39 reported studies pooled according to different factors[9–15,18,19,25–54]*

	# Studies pooled	Extrusions	total tubes	Extrusion rate %	Standard deviation	95% Confidence interval
Adults	19	37	1505	2.5	2.31	0.033
Children	12	26	467	5.6	1.99	0.036
Mixed population	8	14	1071	1.3	2.05	0.045
Total	39	77	3043	2.5	2.11	0.021
Patch	33	69	2654	2.5	2.19	0.024
Scleral flap	4	8	249	3.2	1.41	0.044
No-patch	2	0	40	0.0	0	N/A
No-Patch plus our series	3	0	168	0	0	N/A
Mitomycin C in all or some	7	23	1065	2.2	1.99	0.047
No-mitomycin C	32	54	1978	2.7	2.28	0.025

* For full list of the additional 31 references we used, please email the corresponding author

Furthermore, we have not found dyscoria related to the tube in this series, as reported by others.[15] We feel this low incidence of dyscoria is probably related to a smaller entrance and less flow of aqueous around the tube, which is related to the long episcleral tunnel; this makes it less likely for the iris to be trapped at the entrance site.

Initial visual acuity was not a prognostic factor for failure in our series, although all patients (except one with 20/200 vision) with loss of light perception had worse than counting fingers at 3 m, before surgery. This particular eye with better vision developed a blebitis and subsequent endophthalmitis at the old mitomycin C trabeculectomy site, and eventually required evisceration.

The results in cases with uncertain light perception before surgery are interesting. Of the 15 eyes identified as uncertain, only four were actually demonstrated to be blind after surgery, the rest retained or regained some vision. This could be due to the intensity of examination postoperatively, and / or to better cooperation from the patients as a result of further interaction with the examiner and less photophobia.

Our results are comparable to the previous studies in all other aspects, with similar medium-term results, using strict failure criteria. They clearly show how complications significantly affect the final outcome, making their prevention paramount. Choosing a superotemporal location seems a small and feasible part of the prevention to decrease the risk of complications. On the other hand, eyes with previous glaucoma surgeries may have had worse prognosis due to conjunctival scarring, but as only three eyes had previous conjunctival-sparing procedures it was not possible to test this hypothesis.

Limitations of the present study were inherent to its retrospective nature based on a chart review, and the results should be extrapolated with care, especially since most of the variables tested were not hypothesized prior to the analysis. The IOP and visual acuity were not assessed at formal intervals in a masked manner, and some of the IOP’s had to be measured with a Schiotz tonometer.

The final clinical result for control of the glaucoma is not really shown in this kind of study, where failures of the primary procedure cut short the follow-up period. The actual scenario is more along the lines of additional surgeries, procedures, and medications, to maintain a controlled IOP that can preserve vision, such as, additional valves or multiple removals of the fibrous tissue surrounding the valves. Considering the independence that any remaining vision along with modern visual rehabilitation techniques provides to some of the children and the potential role of emerging technologies that might improve this remnant, it seems worthwhile to offer a good chance to preserve nerve fibers for the long years ahead, for this special group of patients.

In conclusion, Ahmed valve implantation without a tube-covering patch is a very safe technique in terms of preventing extrusions and tube exposures, without compromising on the expected success rate in children with different types of glaucoma.
